# Supplementary material for: Genetic Mapping of Genotype-by-Ploidy Effects in Arabidopsis thaliana
Source: Genes (Basel). 2023 May 26;14(6):1161. doi: 10.3390/genes14061161 (PMC10298593; doi:10.3390/genes14061161)
Supplement: Supplementary file 1 [file genes-14-01161-s001.zip › Supplemental figures.pdf]

Genetic mapping of genotype-by-ploidy effects in *Arabidopsis thaliana*

Cris L. Wijnen[1,2], Frank F.M. Becker[1], Andries A. Okkersen[1], C. Bastiaan de Snoo[3], Martin P. Boer[2], Fred A. van Eeuwijk[2], Erik Wijnker[1], Joost J.B. Keurentjes[1]

[1] Laboratory of Genetics, Wageningen University & Research, Droevendaalsesteeg 1, 6708 PB, Wageningen, The Netherlands. [2] Biometris, Wageningen University & Research, Droevendaalsesteeg 1, 6708 PB, Wageningen, The Netherlands. [3] Rijk Zwaan R&D Fijnaart, Eerste kruisweg 9, 4793 RS Fijnaart, The Netherlands.

Supplemental figures

Supplemental Figure S1: Genetic maps of a DH and F_2 population of a cross between T540 and Ge-0.
Supplemental Figure S2: Allele frequency distributions of markers in the DH and F_2 populations.
Supplemental Figure S3: Heritabilities of morphological traits and correlation between them.
Supplemental Figure S4: Genotype specific variation in response to differences in ploidy level.
Supplemental Figure S5: Genomic sequence variation at the *VIN3* locus.
Supplemental Figure S6: Genomic sequence variation at the *TFL1* and *ARF2* loci.
Supplemental Figure S7: Examples of monoploid plants.

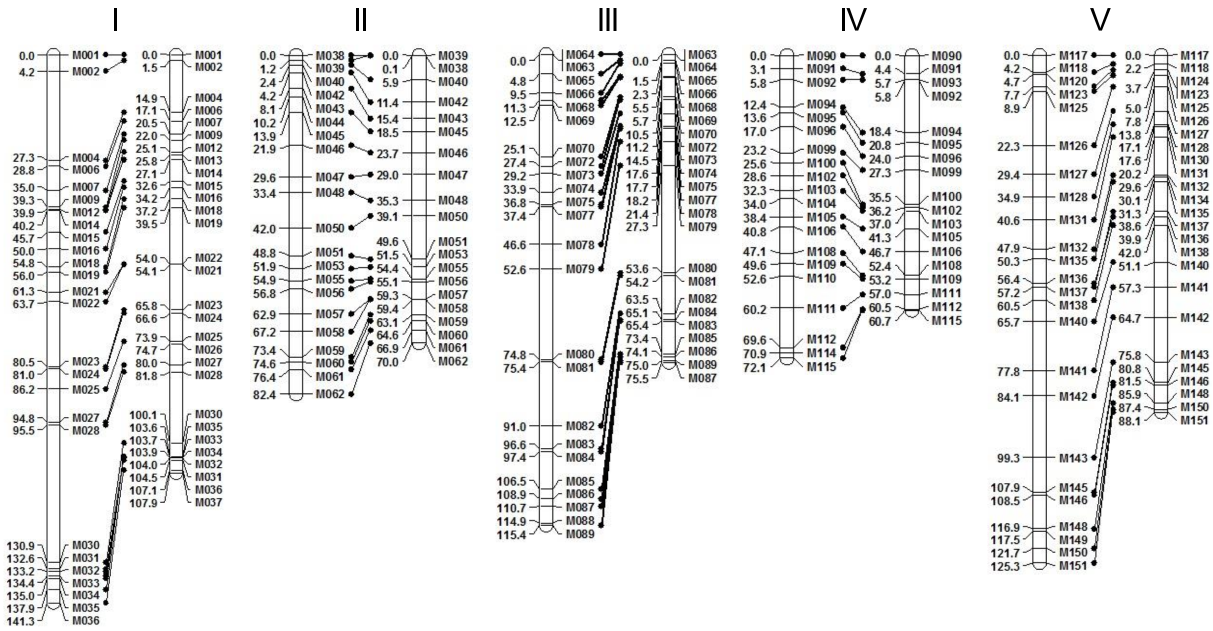

Supplemental Figure S1: Genetic maps of a DH and F_2 population of a cross between T540 and Ge-0.

Chromosome numbers are indicated on the top. Marker positions are indicated in cM at the left of the chromosome bars, marker IDs on the right side. Maps of the DH population (left bar) are aligned to maps of the F_2 population (right bar) by shared markers.

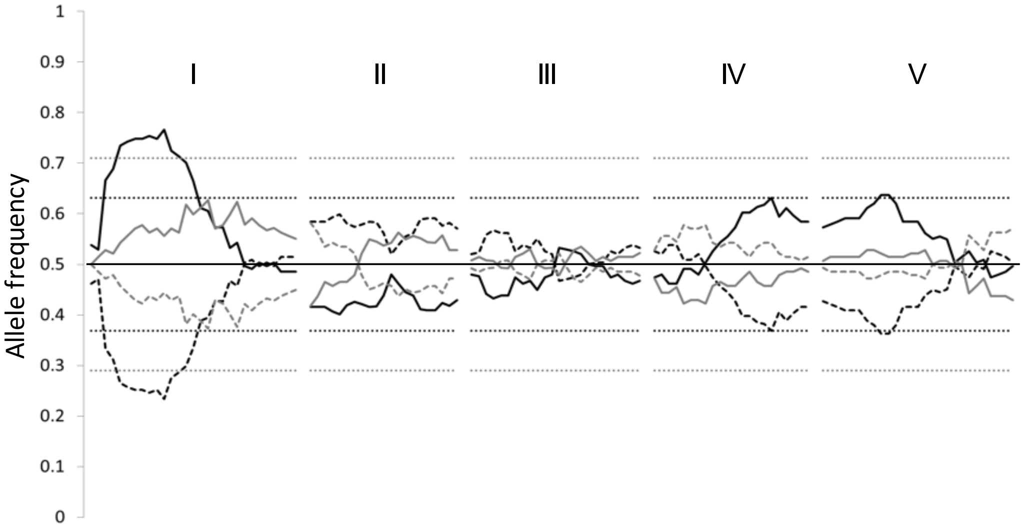

Supplemental Figure S2: Allele frequency distributions of markers in the DH and F_2 populations. Chromosomes are separated by spacing and chromosome numbers are indicated on the top. Black and grey solid lines denote T540 marker allele frequencies in the DH and F_2 population, respectively. Black and grey dashed lines denote Ge-0 marker allele frequencies in the DH and F_2 population, respectively. Black and grey dotted horizontal lines represent thresholds for the critical value of X^2-distributions of the DH and F_2 population, respectively.

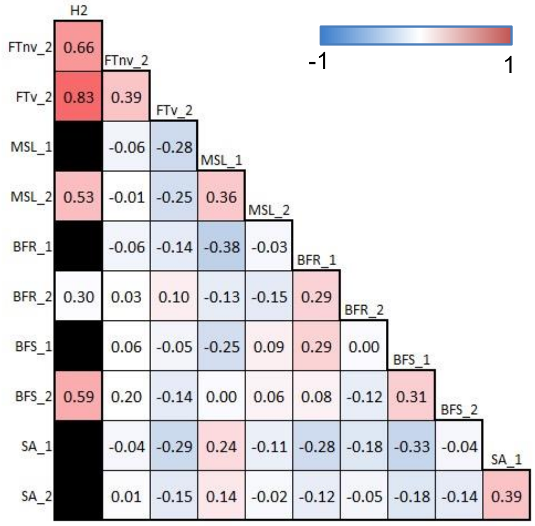

Supplemental Figure S3: Heritabilities of morphological traits and correlation between them. Broad-sense heritabilities (H^2) are listed in the first column. Note that replicated measurements, necessary for calculation of H^2 were only available for the diploid population. Values of Pearson correlation between traits in rows and columns are listed in the half diallel matrix. Correlations of $-0.15 > r^2 > 0.15$ were significant at $\alpha = 0.05$. H^2 = Broad-sense heritability; FTnv = Flowering time without vernalization (days after sowing); FTv = Flowering time after vernalization (days after transfer); MSL = Main stem length (cm); BFR = Branching from rosette (nr.); BFS = Branching from stem (nr.); SA = Seed area (mm^2). The appendix following the trait abbreviations indicates traits measured on the monoploids (-1) or diploids (-2), respectively.

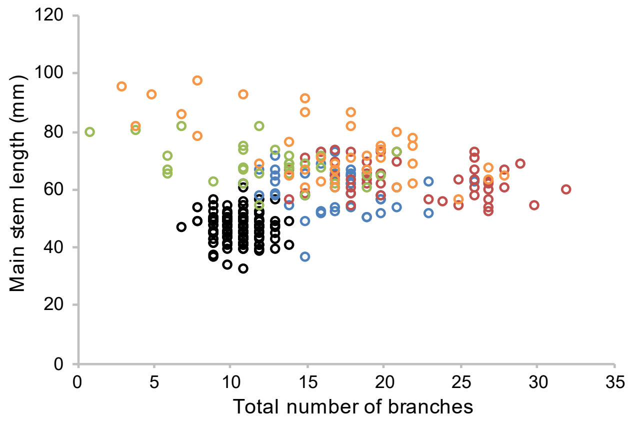

Supplemental Figure S4: Genotype specific variation in response to differences in ploidy level. The total number of branches is the sum of the branches from the rosette and the main inflorescence. Different colours represent different haplotypes based on the genotype at the major QTLs at the top and bottom of chromosome 5, respectively. Black, Diploids; Blue, Monoploid AA; Red, Monoploid AB; Green, Monoploid BA; Orange, Monoploid BB, where A and B represent the T540 and Ge-0 allele, respectively.

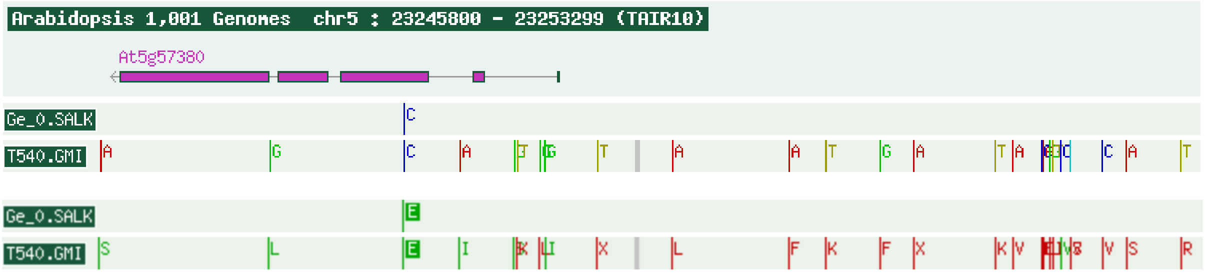

Supplemental Figure S5: Genomic sequence variation at the *VIN3* locus.

Variation in DNA sequence (top) and amino acid sequence (bottom) between Ge-0 and T540. Many SNPs polymorphic between the two accessions result in non-synonymous amino acid substitutions (indicated in red in the bottom panel) in a 3kb upstream region. (Data from the SALK Arabidopsis genome browser (http://signal.salk.edu/)).

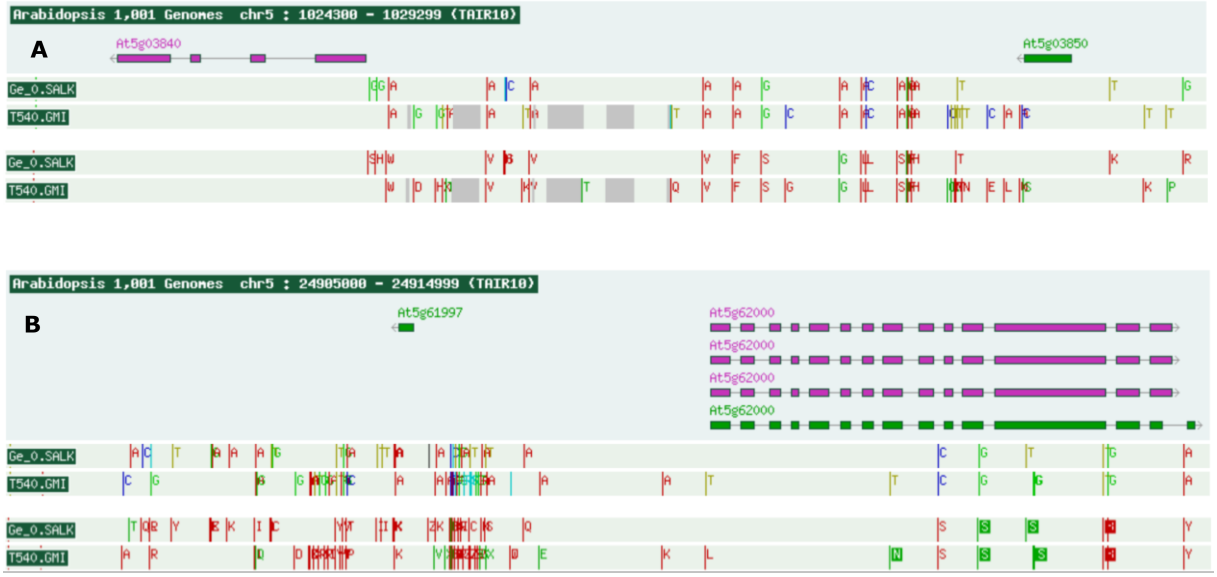

Supplemental Figure S6: Genomic sequence variation at the *TFL1* and *ARF2* loci. Variation in DNA sequence (top) and amino acid sequence (bottom) between Ge-0 and T540. A) The *TFL1* locus is a possible candidate for the QTL on the top of chromosome 5. B) The *ARF2* locus is a candidate gene for the QTL at the bottom of chromosome 5. (Data from the SALK Arabidopsis genome browser (http://signal.salk.edu/)).

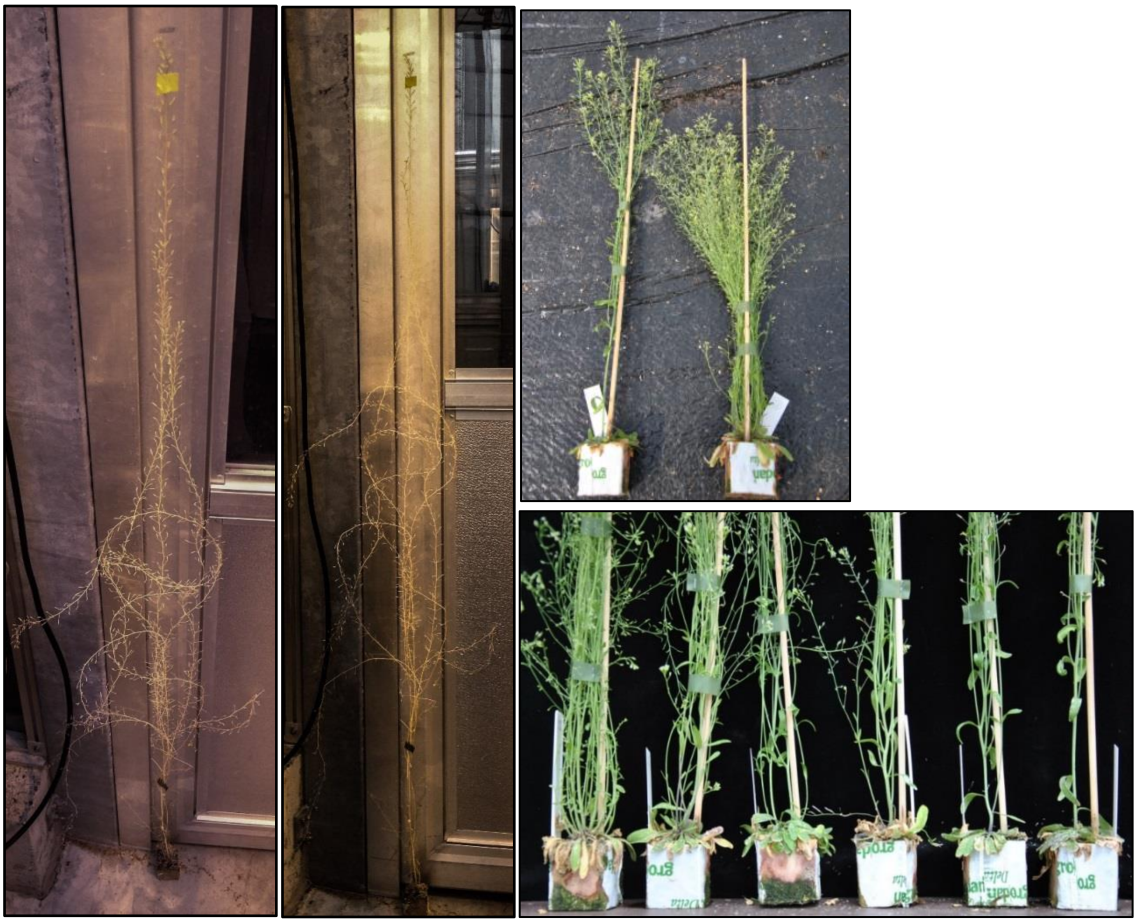

Supplemental Figure S7: Examples of monoploid plants. a-b) The monoploid plant shown is derived from a Col-0 x L*er* F_1. It is shown to represent the extreme plant height monoploids can reach. This specific monoploid stands in a rockwool block of 4 x 4 cm and has a total length of 143 cm. **c-d)** A set of monoploids derived from T-540 x Ge-0 that show variation in the shoot architecture. Note the difference in length (c) and the different number of shoot branches (d). All monoploids are sterile and as such do not produce seed-filled siliques.
